# The Effect of Classical Music on Heart Rate, Blood Pressure, and Mood

**DOI:** 10.7759/cureus.27348

**Published:** 2022-07-27

**Authors:** Cyrus Darki, Jennifer Riley, Dina P Dadabhoy, Amir Darki, Jennifer Garetto

**Affiliations:** 1 Science, The Avery Coonley School, Downers Grove, USA; 2 Literacy, The Avery Coonley School, Downers Grove, USA; 3 Rheumatology, Northwest Rheumatology Specialists, Elk Grove, USA; 4 Interventional Cardiology, Loyola University Medical Center, Maywood, USA

**Keywords:** autonomic nervous system, mood and anxiety, blood pressure variability, heart rate recovery, classical music

## Abstract

Anxiety and depression have deleterious effects on health. Numerous studies have demonstrated the negative impact of emotions such as stress and anxiety on heart rate (HR), blood pressure (BP), and heart disease. These mood states have been linked to stroke, heart failure, diabetes, heart disease, respiratory problems, and drug abuse. Negative emotions can affect the HR and BP through the link between the nervous system and the cardiovascular system. Our study demonstrates the positive effect of classical music on HR, BP parameters, and mood states.

## Introduction

Anxiety and depression have deleterious effects on health. Several studies have demonstrated the negative impact of emotions such as stress and anxiety on heart rate (HR), blood pressure (BP), and heart disease [1]. These mood states have been linked to stroke, heart failure, diabetes, heart disease, respiratory problems, and drug abuse [1]. Negative emotions can affect the HR and BP through the link between the nervous system and the cardiovascular system [2].

Increasing evidence has linked the beneficial effect of music and managing anxiety and depression. Cherry concluded that the psychological effects of music can be powerful and wide-ranging including improving cognitive performance, reducing stress, improving athletic performance, and enhancing sleep [3]. Gold et al. illustrated that those subjects who had musical exposure were able to more effectively complete tasks as compared to those not exposed [4].

Even though there is evidence to support the positive effect of music on HR, BP, and mood there are many inconsistencies in prior studies driven by heterogeneity and small sample sizes. Additional limitations include a lack of a broad range of ages and not including a mood survey.

We aimed to expand on prior studies looking at the effect of music on HR, BP, and mood states utilizing two classical pieces, “Symphony of Fate” by Beethoven and “Moonlight Sonata” by Beethoven.

## Materials and methods

Materials

For the study, we utilized wireless headphones, Kardia electrocardiography system, autonomic blood pressure cuff, fast song: Beethoven’s Symphony of Fate, slow song: Beethoven’s Moonlight Sonata, mood survey, and tablet.

A five-questionnaire mood survey was designed by the study authors specifically for this study.

Procedure

After participants were briefed about the study and consent was obtained, subjects sat for one minute and then asked for his/her age, gender, if he/she enjoys classical music, takes any medications, and is a musician. Then the resting heart rate and blood pressure of the subject were taken using an EKG system and a blood pressure monitor. The subject then listened to a fast classical piece (first movement of Symphony of Fate). The heart rate was recorded on the EKG system 40 seconds into the song. The blood pressure of the same subject was recorded after the performance. The study subject then completed a mood survey followed by a minute break to allow the heart rate and blood pressure to normalize.

Next, the subject listened to a slow classical piece (Moonlight Sonata 1). The heart rate was checked on the EKG system 90 seconds into the song. The blood pressure of the same subject was recorded after the performance. The study subject then completed a mood survey.

Data analysis

The mean heart rate, systolic blood pressure, and diastolic blood pressure were calculated using a two-tailed t-test. Statistical significance was defined by a p-value <0.05. Excel (Microsoft® Corp., Redmond, WA) and SPSS 28.0 software (IBM Corp., Armonk, NY) were utilized for statistical analysis.

Subgroup analysis

Subgroup analysis was performed by age stratification (<25, 25 to 55, >55 years old), gender, and if a participant is a musician. All data was stored confidentially. Patient names were de-identified and listed as numerical values.

The study was approved by The Avery Coonley School institutional ethics committee, Downers Grove, Illinois.

## Results

A total of 100 participants were enrolled in the study. There were 53 males and 47 females. The mean age for participants was 39.8 years old +/- 17.8. Forty percent of the participants were musicians. Thirty-five percent of the subjects were on medications. Sixty-two percent of participants enjoyed classical music.

Subjects had a mean resting HR of 75.7 +/- 17.8 beats per minute, mean resting systolic blood pressure of 116.0 +/- 10.9 millimeters of mercury, mean resting diastolic blood pressure of 73.15 +/- 10.0 millimeters of mercury, and a mean arterial pressure of 87.5 +/- 9.4 millimeters of mercury. With fast music, the mean heart rate was 83.0 +/- 11.9 beats per minute, mean systolic blood pressure of 122.1 +/- 13.9 millimeters of mercury, and a mean diastolic blood pressure of 79.7 +/- 11.2 millimeters of mercury. For slow music, the mean systolic blood pressure was 110.5 +/- 9.7 millimeters of mercury, and a mean diastolic blood pressure of 70.7 +/- 9.8 millimeters of mercury. The mean difference in resting, fast music, and slow music heart rate, systolic blood pressure, and diastolic blood pressure was statistically significant (p= <.05, Table 1).

**Table 1:** Mean heart rate, systolic blood pressure, diastolic blood pressure, associated standard deviation, and p-values (p-value ≤ .05 was considered statistically significant).

	Resting	Fast Music	Slow Music	P-Value
Heart Rate (BPM)	75.7 +/- 11.6	83.0 +/- 11.9	72.6 +/- 11.3	<0.05
Systolic Blood Pressure (mmHg)	116.0 +/- 10.9	122.1 +/- 13.9	110.5 +/- 9.7	<0.05
Diastolic Blood Pressure (mmHg)	73.2 +/- 10.1	79.7 +/- 11.2	70.7 +/- 9.8	<0.05

**Figure 1:**
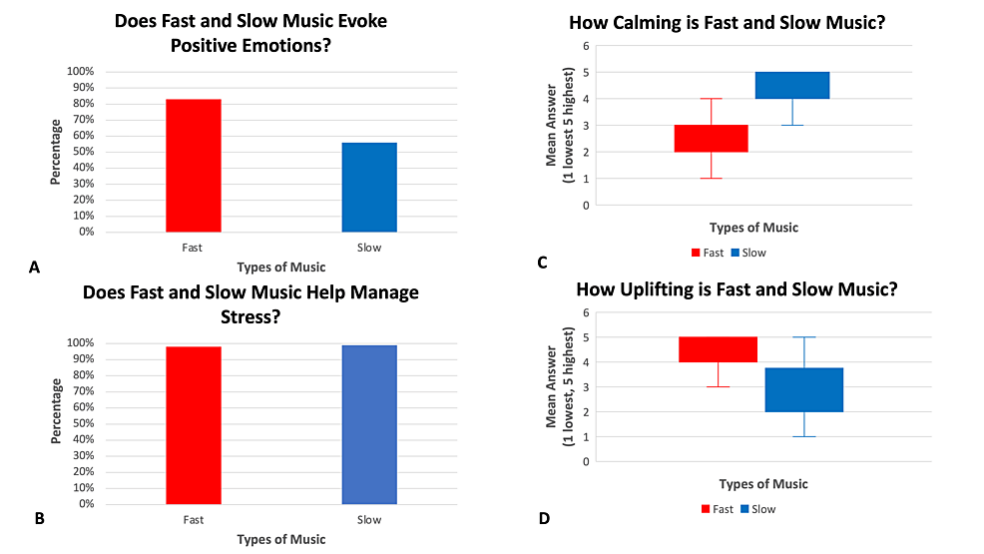
The Effect of Fast and Slow Music on Emotion and Stress. (A) Does fast and slow music evoke positive emotions? (B) Does fast and slow music help manage stress? (C) How calming is fast and slow music? (D) How uplifting is fast and slow music?

All 100 subjects completed a mood survey using a scale of 1-5 (1 being lowest, 5 being highest). The mean score for mood survey Q1 “how uplifting the song was” was 4.2 +/- 1.0 for fast music and 2.9 +/- 1.0 for slow music (Figure 1). The mean score for mood survey Q2 “how calming the song was” was 2.8 +/- 0.8 for fast music and 4.5 +/- 0.8 for slow music (Figure 1). A total of 83% of subjects reported that fast music created positive emotions while 56% of subjects reported that slow music created positive emotions. Three percent of subjects said that fast music created negative feelings and 9% of subjects said that slow music created negative emotions. A total of 98% of participants said that fast music helps manage stress and 99% of participants said that slow music helps manage stress (Figure 1).

To further analyze the data, subjects were categorized into the following age groups: <25, 25 to 55, and >55 years old. There were 16 subjects in the group <25 years old, 66 subjects in the group 25 to 55 years old, and 18 subjects in the group >55 years old. The data was also stratified by gender and whether the participants were musicians.

The mean resting heart rate for <25, 25 to 55, and >55 years old groups were 78.5 +/- 29.0 beats per minute, 75.1 +/- 17.0 beats per minute, and 75.7 +/- 13.2 beats per minute. After listening to fast music, the heart rate for <25, 25 to 55, and >55 years old was 80.6 +/- 12.0 beats per minute, 80.3 +/- 11.7 beats per minute, and 83.7 +/- 13.0 beats per minute, respectively. After listening to slow music, the heart rate for <25, 25 to 55, and >55 years old was 81.6 +/- 12.4 beats per minute, 70.8 +/- 10.8 beats per minute, and 71.1 +/- 8.7 beats per minute. There was no statistical difference between the resting and fast heart rate groups. Slow music heart rate, however, was statistically significantly lower for ages 25 to 55 and >55 as compared to <25 years old (p = .002).

**Figure 2:**
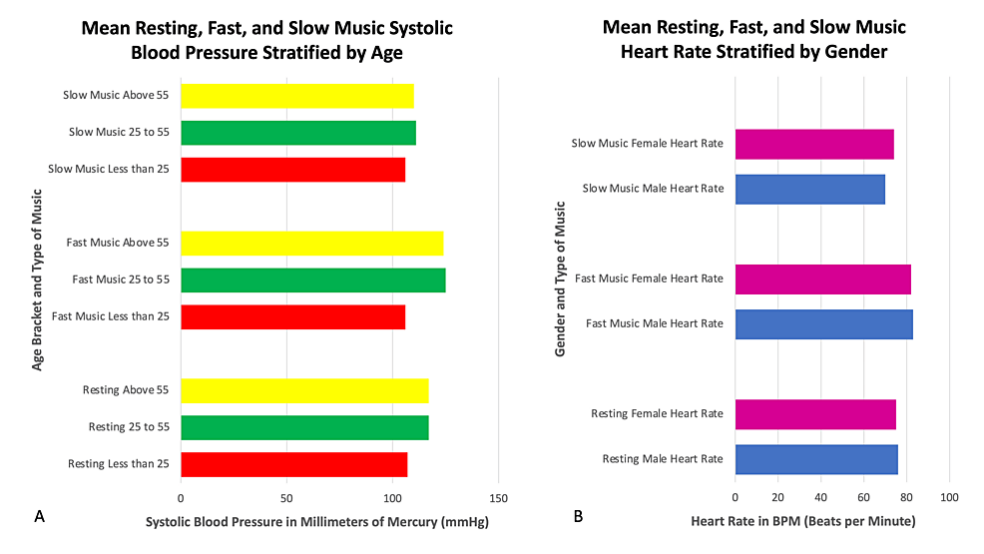
Effect of Music on Blood Pressure and Heart Rate Stratified by Age and Gender. (A) The mean resting, fast, and slow music systolic blood pressure stratified by age. (B) The mean resting, fast, and slow music heart rate stratified by gender.

The resting systolic blood pressure for <25, 25 to 55, and >55 years old was 107.0 +/- 10.7 millimeters of mercury, 117.8 +/- 10.5 millimeters of mercury, and 117.7 +/- 13.2 millimeters of mercury. After listening to fast music, the systolic blood pressure for <25, 25 to 55, and >55 years old increased to 106.6 +/- 12 millimeters of mercury, 125.6 +/- 11.7 millimeters of mercury, and 124.3 +/- 10.8 millimeters of mercury, respectively. After listening to slow music, the systolic blood pressure for <25, 25 to 55, and >55 years old decreased to 106.2 +/- 8.7 millimeters of mercury, 111.6 +/- 9.5 millimeters of mercury, and 110.4 +/- 10.8 millimeters of mercury. With regards to systolic blood pressure, those subjects <25 years old had a statistically lower systolic blood pressure as compared to the other two groups (Figure 2, p = .001). This difference was maintained with fast music (Figure 2, p = .001). With slow music, there was no statistical difference in the three groups (Figure 2).

The resting heart rate for males and females was 76.2 +/- 11 beats per minute and 75.1 +/- 17.0 beats per minute. After listening to fast music, the heart rate for male and female was 80.6 +/- 12.0 beats per minute and 80.3 +/- 11.7 beats per minute, respectively. After listening to slow music, the heart rate for male and female was 81.6 +/- 12.4 beats per minute and 70.8 +/- 10.8 beats per minute, respectively. There were no statistically significant gender differences in resting heart rate, systolic blood pressure, and diastolic blood pressure. The resting systolic blood pressure for males and females was 115.2 +/- 10.7 millimeters of mercury and 117.1 +/- 11.2 millimeters of mercury. After listening to fast music, the systolic blood pressure for male and female was 121.5 +/- 13.9 millimeters of mercury and 122.4 +/- 14.0 millimeters of mercury, respectively. After listening to slow music, the systolic blood pressure for male and female was 111.0 +/- 8.8 millimeters of mercury and 110.2 +/- 10.8 millimeters of mercury, respectively. There were no statistically significant gender differences in resting heart rate, systolic blood pressure, and diastolic blood pressure (Figure 2).

The resting heart rate for musicians and non-musicians was 75.8 +/- 11.9 beats per minute and 75.7 +/- 11.4, respectively. After listening to fast music, the heart rate for musicians and non-musicians was 82.2 +/- 12.7 beats per minute and 84.1 +/- 10.7 beats per minute, respectively. After listening to slow music, the heart rate for musician and non-musician was 73.1 +/- 11.3 beats per minute and 71.9 +/- 11.4 beats per minute, respectively. The resting systolic blood pressure for musicians and non-musicians was 118.2 +/- 10.4 millimeters of mercury and 112.8 +/- 10.9 millimeters of mercury, respectively. After listening to fast music, the systolic blood pressure for musicians and non-musicians was 122.9 +/- 13.5 millimeters of mercury and 120.9 +/- 14.5 millimeters of mercury, respectively. After listening to slow music, the systolic blood pressure for musicians and non-musician was 112.0 +/- 10.4 millimeters of mercury and 108.3 +/- 8.2 millimeters of mercury, respectively. Musicians had a statistically significant lower resting systolic blood pressure and lower heart rate after slow music as compared to non-musicians (p-value = .001).

## Discussion

The major findings of this study include: one, listening to fast music increased heart rate, systolic, and diastolic blood pressure; two, listening to slow music decreased heart rate, systolic, and diastolic blood pressure; three, mood survey scores were favorable for both fast and slow music. Fast music was viewed as uplifting with a mean score of 4.2 +/- 1.0 (out of 5). Listening to slow music was viewed as calming with a mean score of 4.5 +/- 0.8 (out of 5). For fast and slow songs, 98% and 99% of subjects reported that the music could help manage stress respectively. The mean difference in resting, slow, and fast heart rate, systolic blood pressure, and diastolic blood pressure were all statistically significant.

To further analyze the study data, the following subgroup analysis was completed: age stratification (<25, 25-55, >55 years old), gender differences, and whether subjects were musicians. Subjects <25 years old had a statistically lower resting systolic blood pressure as compared to the other two groups (p = .001). Listening to slow music reduced all systolic blood pressures highlighting the calming effect of slow music and the therapeutic potential for slow, classical music.

The study did not identify gender differences in resting heart rate, systolic, and diastolic blood pressure, emphasizing the uniform benefit of music in both males and females. Musicians did have a statistically significantly lower systolic blood pressure after listening to music (p = .001) possibly related to pleasant memories from prior musical experiences.

The physiologic changes in heart rate and blood pressure while listening to fast and slow music are complex. Music affects the cardiovascular system through multiple potential mechanisms. One pathway includes brain signals responding to music rhythms through signal activations to organs of the body, including the heart, which then respond to the tempo of the song -- i.e., when the tempo is fast, the heart rate and blood pressure speed up, and when the tempo is slow the heart rate and blood pressure slow down [5]. Similar to the findings in this study, Suguna and Deepika reported that fast music increases heart rate and blood pressure, and slow music decreases both parameters [6]. Furthermore, Bernardi et al. observed that fast-beat music has an arousal effect proportional to the speed of music [7].

Another pathway explaining the effect of music on the cardiovascular system is the role of the autonomic system. Ellis and Thayer described how heart rate is under the control of the parasympathetic nervous system through the vagus nerve [8, 9]. The vagus nerve, cranial nerve X, is located near the eardrum and responds to musical vibrations by triggering the body to relax. This pathway may explain the study observations which found lower systolic blood pressure after listening to slow, classical music [10].

Music also affects other parts of the brain, which in turn affects the mood through the release of neurotransmitters such as dopamine. Ellis and Thayer described the release of dopamine from the nucleus accumbens after listening to pleasurable, classical music [8]. Salimpoor et al., using magnetic resonance imaging, demonstrated dopamine release at the peak of emotional arousal during music listening [11]. Dopamine release may contribute to the study findings which found that 83% of subjects found fast music uplifting. In the subjects that did not find the classical music pieces uplifting, Koelsch and Jäncke described that different tastes and preferences of music may affect people’s response to a certain piece [1].

Finally, nearly all subjects in the study found that music can help manage stress. This has been previously reported. Agrawal et al. demonstrated that people use music as a tool to improve their emotions or their athletic performance [5]. Additionally, McCraty et al. found that classical music, in general, has many benefits including the reduction in anxiety and depression [2, 12-13].

The study has several important limitations. First, there were fluctuations in the testing environment and in the subjects’ baseline stress levels. For example, some subjects were not comfortable being tested inside due to the COVID-19 pandemic which resulted in varying conditions that could potentially affect outcomes. Also, subjects who entered the study with higher levels of stress may have experienced a higher heart rate and blood pressure than those who were more relaxed. Second, while the study enrolled 100 subjects, the standard deviations of approximately +/- 10 in each cohort can be improved by a larger sample size. Additionally, only 16% of subjects were under the age of 25 years old and 18% >55 years old. There were no significant outliers in the study.

## Conclusions

In conclusion, our study suggests classical music has a positive impact on the cardiovascular system and potential emotional benefits. Music affects the cardiovascular system through multiple potential mechanisms including the autonomic nervous system and the vagus nerve which responds to musical vibrations by triggering the body to relax. Music also affects other parts of the brain, which in turn affects the mood through the release of neurotransmitters such as dopamine. Dopamine release may contribute to the study findings which found that 83% of subjects found fast music uplifting. Finally, nearly all subjects believe music can help manage stress. Listening to music may be a potential therapeutic method for reducing anxiety and depression. Given the large sample size, the study adds greatly to the current literature by validating the results of other smaller studies.
